# Real‐World Performance of FIT Triage for Symptomatic Colonoscopy: Analysis of the UK National Endoscopy Database (NED)

**DOI:** 10.1111/apt.70537

**Published:** 2026-01-28

**Authors:** David Beaton, Mahmoud Mohamed, Linda Sharp, Keith Pohl, Matthew Rutter

**Affiliations:** ^1^ Northumbria NHS Foundation Trust North Shields UK; ^2^ Population Health Sciences Institute, Newcastle University Centre for Cancer, Newcastle University Newcastle‐upon‐Tyne UK; ^3^ North Tees and Hartlepool NHS Foundation Trust Stockton‐on‐Tees UK; ^4^ Torbay and South Devon NHS Foundation Trust Torquay UK

## Abstract

**Background:**

The UK has adopted faecal immunochemical testing (FIT) to triage symptomatic colonoscopy referrals.

**Objective:**

Quantify diagnostic yield and independent effects of FIT, age, sex and symptoms on polyp and cancer detection in symptomatic colonoscopy.

**Design:**

Nationwide analysis of prospectively collected colonoscopy reports (June 2023–August 2025) from the National Endoscopy Database. Symptomatic procedures, including iron‐deficiency anaemia (IDA), were identified and diagnostic yields calculated. Mixed‐effects logistic regression estimated adjusted odds ratios (aORs) with age, sex, symptom group and FIT. Post‐estimation margins modelled cancer yield by age, FIT and symptoms.

**Results:**

Analysis of 447,109 symptomatic colonoscopies, with FIT recorded in 202,219 (45.2%). Overall cancer yield was 1.9% (95% CI, 1.8–1.9).

Cancer yield was 3.8% (95% CI, 3.7–3.9) in FIT ≥ 10, including 2.5% (95% CI, 2.3–2.7) at age 40–49 and 0.6% (95% CI, 0.5–0.7) at age 16–39; yield in FIT < 10 was 0.3% (95% CI, 0.2–0.3).

FIT concentration showed a strong, graded association with cancer risk, weaker association with large polyps, and minimal association with small polyps. IDA was associated with higher cancer risk versus rectal bleeding (aOR 2.2, 95% CI, 2.0–2.3; *p* < 0.01).

With FIT < 10, cancer yield exceeded 1% only in IDA patients aged > 80 and remained < 0.5% otherwise. A model combining FIT, age, and IDA detected > 94% of cancers while reducing colonoscopy demand by > 40%.

**Conclusion:**

This national analysis demonstrates the superiority of FIT‐based triage over symptom‐based referral, with FIT ≥ 10 identifying high‐risk patients, including those aged 40–49, while FIT < 10 indicated very low risk.

## Graphical abstract

National endoscopy data show that FIT‐based triage clearly outperforms symptom‐based referral for colorectal cancer risk stratification. FIT ≥ 10 identifies high‐risk symptomatic patients, while FIT < 10 confers very low cancer risk, supporting FIT as the primary tool for prioritising colonoscopy in symptomatic pathways.

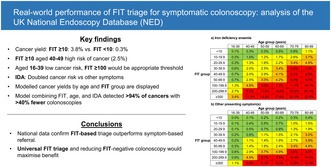

## 1 Introduction

Colorectal cancer (CRC) is the second most common cause of cancer‐related deaths worldwide [1] with over 16,000 deaths annually in the United Kingdom (UK) alone [2]. Colonoscopy remains central to the diagnosis of CRC, enabling direct mucosal visualisation alongside histological sampling. In addition, it offers a preventive role through the detection and removal of precancerous polyps, reducing future CRC risk [3].

Most UK colonoscopies are undertaken to investigate lower gastrointestinal symptoms [4], yet many symptoms have high prevalence in the general population [5] and investigation yields low rates of CRC or advanced pathology [6]. To reduce low‐yield investigation and ease service pressures, the National Institute for Health and Care Excellence (NICE) and the British Society of Gastroenterology recommend the use of faecal immunochemical testing (FIT) in patients with symptoms of suspected CRC [7, 8]. A threshold of fHb ≥ 10 μg Hb/g was recommended to guide urgent referral for colonoscopic investigation [8], supported by systematic review and meta‐analysis data demonstrating 87%–92% sensitivity and 84%–88% specificity at this cut‐off [9].

Despite high sensitivity and specificity at the ≥ 10 μg Hb/g threshold, concerns persist regarding potential missed pathology [10]. Recent data also suggests practitioners have concerns about overuse of FIT and the burden on endoscopy services of investigations for patients with false‐positive results [11]. Alongside this, initial guidance only proposed a single FIT threshold (≥ 10), with insufficient evidence available to further stratify risk based on patient factors such as age, sex and presenting symptom.

The National Endoscopy Database (NED) captures data from electronic endoscopy reports from the majority of units across the UK, uploaded automatically in near real time from participating centres [12]. This provides a unique national dataset to assess the impact of symptomatic FIT on colonoscopy yield and to explore potential refinements to referral pathways.

The primary aim of this study was to evaluate the diagnostic yield of colonoscopy in symptomatic patients following the introduction of FIT and to examine how additional patient factors influence pathology risk, with the goal of improving risk stratification and refining referral pathways.

## 2 Methods

### 2.1 Data Source and Study Population

Prospectively collected colonoscopy data for procedures performed in the UK between June 2023 and August 2025 (27 months) were extracted from the second iteration of the National Endoscopy Database (NEDi2), which includes FIT result as a mandatory field. By August 2025, 412 sites (73% of UK endoscopy units) had uploaded to NEDi2 [13]. Further details of NEDi2 are available on the NED webpage [13], while the process for automatic upload of electronic endoscopy reports to NED has been described previously [12]. Data from all colonoscopies uploaded to NEDi2 during this period were extracted.

**FIGURE 1:**
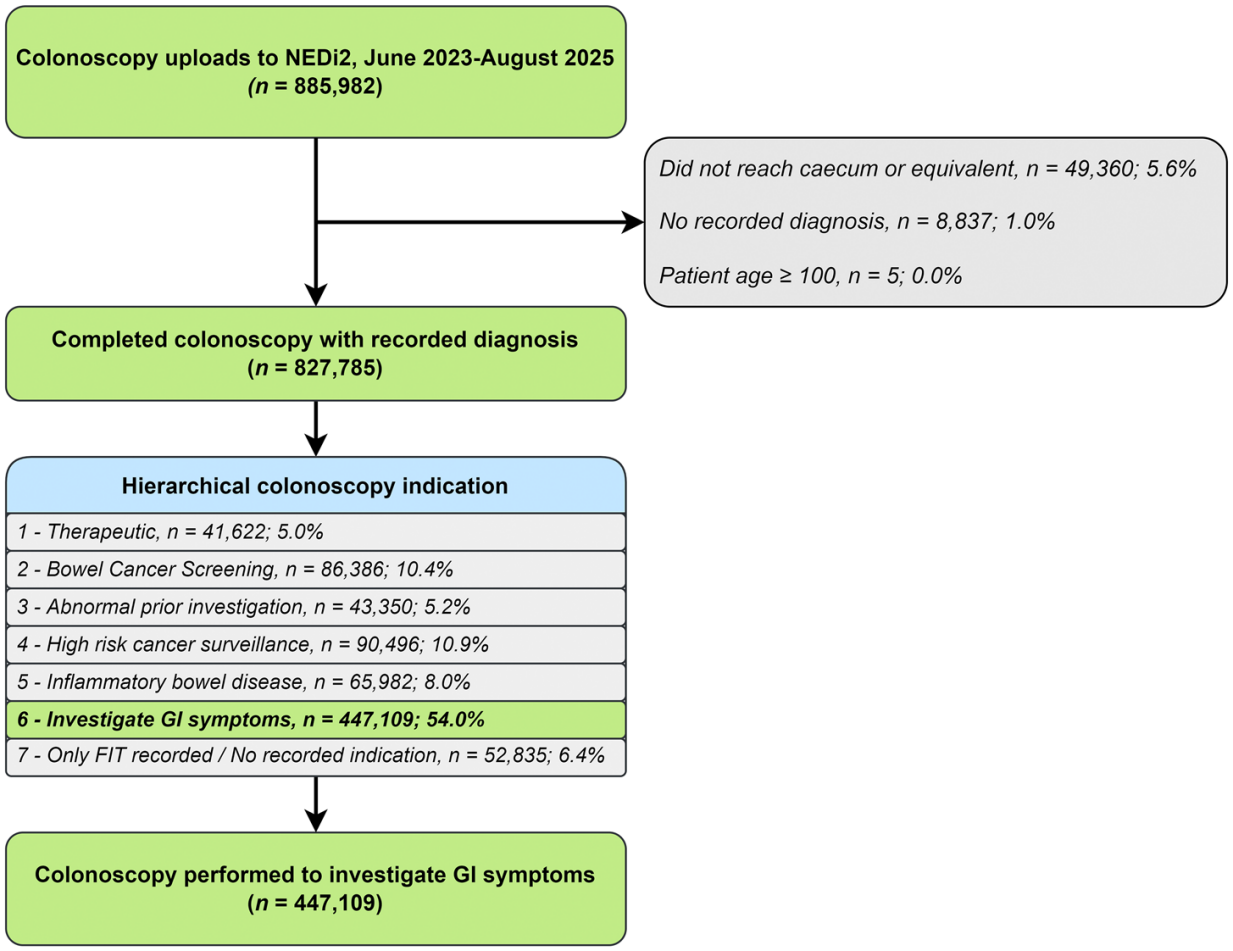
Flow diagram of study inclusion and exclusion criteria. NEDi2, second iteration of National Endoscopy Database; GI, gastrointestinal.

Procedures were excluded if caecal (or equivalent) intubation was not achieved (except when incomplete intubation was due to malignancy), if no diagnosis was recorded, or if patient age was recorded as 100 or older (Figure 1).

Structured and free‐text indication and diagnosis fields were consolidated using case‐insensitive keyword searches, with full mappings detailed in Table S1. As CRC was a key outcome, all free‐text entries flagged by keyword matching were manually reviewed prior to assignment of a CRC diagnosis.

This analysis focused on colonoscopies performed to investigate gastrointestinal symptoms, alongside indications of anaemia and iron‐deficiency anaemia (IDA), hereafter referred to as symptomatic colonoscopies. A hierarchical classification (Figure 1) was applied to ensure inclusion was restricted to these procedures, excluding colonoscopies performed for alternative indications, including: surveillance, screening, inflammatory bowel disease (IBD) assessment or surveillance, raised faecal calprotectin, abnormal prior investigation, planned therapy, or where no indication was recorded.

### 2.2 Categorisations

Symptomatic colonoscopies were categorised into mutually exclusive symptom groups, based on a hierarchy of symptoms determined using previously defined methodology [6]. Specifically, for procedures with multiple recorded symptoms, the indication with the highest priority in the hierarchy was assigned (e.g., a case with both IDA and constipation was classified as IDA).

As the NEDi2 dataset is anonymised and not linked to histology, diagnoses were based on the endoscopist's reported findings. Each procedure was assigned to a single, mutually exclusive hierarchical diagnosis group, prioritised by clinical severity: CRC > large polyp (≥ 10 mm) > small polyp (< 10 mm) > other diagnosis > haemorrhoids > normal/diverticulosis. The classification process, alongside classification of diagnosis groups and free‐text matching, is detailed in Table S1.

FIT results on NEDi2 were recorded either as a string (e.g., ‘FIT positive’, ‘FIT negative’, ‘FIT not performed’) or as a numeric value. Numeric values were capped at 400 and grouped as: < 10, 10–19, 20–29, 30–39, 40–49, 50–99, 100–149, 150–199, 200–299 and ≥ 300. The analysis category of ‘FIT < 10’ included all numeric results < 10 and ‘FIT negative’, while the category of ‘FIT ≥ 10’ included all numeric results ≥ 10 and ‘FIT positive’. Cases without a recorded FIT result (including ‘FIT not done’ and ‘FIT awaited’) were classified as ‘FIT not recorded’. As NED data are anonymised and FIT results are derived from the endoscopy report, it was not possible to cross‐reference FIT values with laboratory reporting systems.

### 2.3 Statistical Analysis

Descriptive statistics summarised symptomatic colonoscopy distributions by age group, sex, and hierarchical symptom and diagnosis groups. The prevalence of each was calculated overall and by FIT category (≥ 10, < 10, or not recorded). FIT ≥ 10 and < 10 categories include corresponding numeric results and ‘FIT positive/negative’ entries. The yield of large polyps and CRC was then calculated by patient age‐group for both FIT ≥ 10 and FIT < 10 colonoscopies.

Mixed‐effects logistic regression models were constructed to estimate adjusted odds ratios (aORs) with 95% confidence intervals (CIs) for detection of small polyps (< 10 mm), large polyps (≥ 10 mm), and CRC. Model diagnostics were undertaken to assess collinearity and clustering. Multivariable collinearity was evaluated using variance inflation factors (VIFs), with no evidence of concerning collinearity observed (mean VIF 1.5). Alternative random‐effects structures were explored to account for procedural‐level correlation, including clustering by endoscopist and by site. Endoscopist‐level clustering explained a greater proportion of variation in CRC detection and was therefore retained in the final models. Fixed effects included patient age group, sex (with the small number of ‘unknown’ recategorised as female), hierarchical symptom group and FIT category, with endoscopist entered as a random effect. All estimates were mutually adjusted for all covariates.

Post‐estimation margins were used to model predicted CRC yields across age, sex and FIT group, stratified by IDA versus all other presenting symptom(s) combined. The ‘anaemia’ group was excluded at this point since pathology linkage was unavailable to differentiate unclassified from misclassified IDA. Modelled yields were presented graphically with colour‐coded risk thresholds (< 1%, 1%–3%, 3%–5%, > 5%).

The impact on colonoscopy workload and detection of significant pathology was evaluated by comparing current practice with two alternative strategies: (a) current guidance, investigating only patients with IDA and/or FIT ≥ 10; (b) restricting investigation to patient groups with a modelled CRC risk ≥ 1%. Current practice included all symptomatic colonoscopies with a numerical FIT concentration recorded (as the analysis focussed on yield for different FIT cut‐offs), excluding the ‘anaemia’ group. The ≥ 1% threshold was selected as a pragmatic cut‐off for routine colonoscopy, with some values rounded up to maintain model simplicity. All data cleaning, coding, and statistical analyses were performed using Stata version 15 (StataCorp, College Station, TX, USA).

### 2.4 Patient and Public Involvement

There was no patient or public involvement in this study.

## 3 Results

Between June 2023 and August 2025, 447,109 symptomatic colonoscopies were uploaded to NEDi2 from 412 UK sites. Over one‐third (34.0%) of these were performed in patients aged under 50 years and over half (53.3%) on female patients (Table 1).

**TABLE 1:** Characteristics and diagnostic outcomes of symptomatic colonoscopy, stratified by FIT category (≥ 10, < 10 μg Hb/g, or not recorded).

Characteristic	FIT ≥ 10 μg Hb/g	FIT < 10 μg Hb/g	FIT not recorded	Overall
Total procedure uploads	152,257 (34.1%)	49,962 (11.2%)	244,890 (54.8%)	447,109 (100.0%)
Age group (years)				
16–39	19,527 (12.8%)	5834 (11.7%)	55,472 (22.7%)	80,833 (18.1%)
40–49	21,232 (13.9%)	7047 (14.1%)	42,840 (17.5%)	71,119 (15.9%)
50–59	28,299 (18.6%)	10,603 (21.2%)	48,114 (19.7%)	87,016 (19.5%)
60–69	33,506 (22.0%)	12,908 (25.8%)	46,668 (19.1%)	93,082 (20.8%)
70–79	37,301 (24.5%)	10,966 (22.0%)	39,353 (16.1%)	87,620 (19.6%)
80–99	12,392 (8.1%)	2604 (5.2%)	12,443 (5.1%)	27,439 (6.1%)
Sex				
Female	76,846 (50.5%)	28,470 (57.0%)	132,824 (54.2%)	238,140 (53.3%)
Male	74,677 (49.1%)	21,117 (42.3%)	109,046 (44.5%)	204,840 (45.8%)
Unknown	734 (0.5%)	375 (0.8%)	3020 (1.2%)	4129 (0.9%)
Hierarchical symptom groups				
1—Iron deficiency anaemia	18,394 (12.1%)	9238 (18.5%)	33,981 (13.9%)	61,613 (13.8%)
2—Anaemia	9347 (6.1%)	2252 (4.5%)	11,824 (4.8%)	23,423 (5.2%)
3—Rectal bleeding	52,679 (34.6%)	10,252 (20.5%)	64,057 (26.2%)	126,988 (28.4%)
4—Weight loss	9415 (6.2%)	2615 (5.2%)	10,464 (4.3%)	22,494 (5.0%)
5—Melaena	210 (0.1%)	43 (0.1%)	1265 (0.5%)	1518 (0.3%)
6—Change in bowel habit	28,442 (18.7%)	11,373 (22.8%)	49,186 (20.1%)	89,001 (19.9%)
7—Diarrhoea	14,683 (9.6%)	8861 (17.7%)	40,442 (16.5%)	63,986 (14.3%)
8—Constipation	6288 (4.1%)	1875 (3.8%)	9124 (3.7%)	17,287 (3.9%)
9—Abdominal pain	11,683 (7.7%)	3080 (6.2%)	22,120 (9.0%)	36,883 (8.3%)
10—Defecation disorder	387 (0.3%)	239 (0.5%)	1589 (0.7%)	2215 (0.5%)
11—Bloating	729 (0.5%)	134 (0.3%)	838 (0.3%)	1701 (0.4%)
Hierarchical diagnosis groups				
1—Cancer	5730 (3.8%)	135 (0.3%)	2443 (1.0%)	8308 (1.9%)
2—Inflammatory bowel disease	9889 (6.5%)	1310 (2.6%)	10,327 (4.2%)	21,526 (4.8%)
3—Large polyp (≥ 10 mm)	15,441 (10.1%)	1876 (3.8%)	10,933 (4.5%)	28,250 (6.3%)
4—Small polyp only (< 10 mm)	48,028 (31.5%)	14,412 (28.9%)	60,671 (24.8%)	123,111 (27.5%)
5—Other diagnosis	2008 (1.3%)	426 (0.9%)	2604 (1.1%)	5038 (1.1%)
6—Haemorrhoids	19,665 (12.9%)	6486 (13.0%)	30,356 (12.4%)	56,507 (12.6%)
7—Normal or diverticulosis	51,496 (34.0%)	25,317 (51.0%)	127,556 (52.1%)	204,369 (45.7%)

*Note:* Values are *n* (%). FIT ≥ 10 includes patients with numeric results (*n* = 135,051) or recorded as ‘FIT positive’ (*n* = 17,206); FIT < 10 includes numeric results (*n* = 33,796) or recorded as ‘FIT negative’ (*n* = 16,166). Symptom and diagnosis groups are mutually exclusive and hierarchically assigned.

The most frequent symptom groups were rectal bleeding (28.4%), change in bowel habit (19.9%), diarrhoea (14.3%) and IDA (13.8%).

The overall CRC yield from symptomatic colonoscopies was 1.9% (95% CI, 1.8–1.9). FIT value was recorded in 202,219 (45.2%) reports. Those with FIT ≥ 10 had a CRC yield of 3.8% (95% CI, 3.7–3.9), 12 times greater than the yield of 0.3% (95% CI, 0.2–0.3) from FIT < 10 colonoscopies. Compared with FIT < 10, the FIT ≥ 10 colonoscopies were also more likely to detect IBD (6.5% (95% CI, 6.4–6.6) vs. 2.6% (95% CI, 2.5–2.8)) and large polyps (10.8% (95% CI, 10.7–11.0) vs. 3.8% (95% CI, 3.7–4.0)) (*p* < 0.001 for both).

**FIGURE 2:**
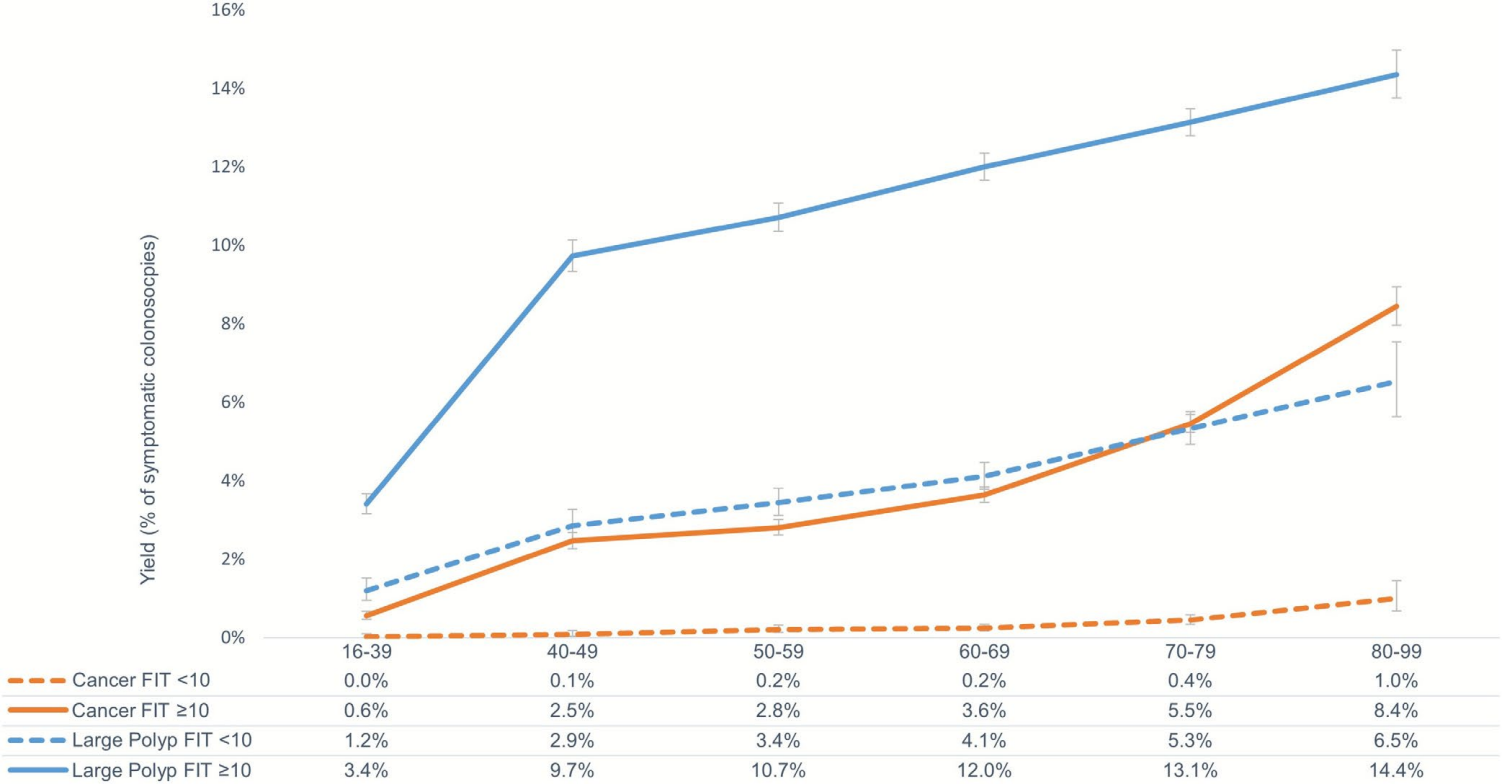
Colorectal cancer and large polyp yield by Age Group and FIT Result: Percentages and 95% confidence intervals.

The yield of CRC and large polyps increased with patient age (Figure 2), with greater yield within each age group, for both CRC and large polyps, in the FIT ≥ 10 group compared to FIT < 10 (*p* < 0.001 for all). CRC yield in those aged 40–49 with FIT ≥ 10 was 2.5% (95% CI, 2.3–2.7), similar to that seen in those aged 50–59 (2.8% (95% CI, 2.6–3.0)) and four times higher than those aged 16–39 (0.6% (95% CI, 0.5–0.7)).

Mixed‐effects logistic regression demonstrated a strong and graded association between FIT group and advanced pathology (Table 2). Compared with FIT < 10, the aOR of CRC rose more than 20‐fold at FIT 100–199 (aOR 21.2 (95% CI, 17.1–26.3)) and over 40‐fold at FIT ≥ 300 (aOR 46.1 (95% CI, 37.3–57.1)). Large polyp detection was also associated with FIT concentration, with odds increasing from aOR 2.2 (95% CI, 2.0–2.4) at FIT 10–19.9 to aOR 3.8 (95% CI, 3.5–4.1) at FIT ≥ 300. By contrast, small polyp detection showed a weaker association with FIT.

**TABLE 2:** Mixed‐effects logistic regression of the association of reporting small polyps, large polyps and colorectal cancer from colonoscopy by patient age group, patient sex, symptoms and FIT group (with endoscopist variation as random effect): Numbers of procedures, adjusted odds ratios (aOR), with 95% confidence intervals (CI) and *p* values.

		Small (< 10 mm) polyp(s)	Large (≥ 10 mm) polyp(s)	Colorectal cancer
Variable	Procedures	aOR (95% CI); *p*	aOR (95% CI); *p*	aOR (95% CI); *p*
Patient age group				
16–39	*20,988*	0.22 (0.21–0.23); < 0.001	0.22 (0.20–0.24); < 0.001	0.08 (0.06–0.10); < 0.001
40–49	*23,605*	0.50 (0.48–0.52); < 0.001	0.68 (0.64–0.72); < 0.001	0.40 (0.36–0.45); < 0.001
50–59	*32,093*	0.66 (0.64–0.69); < 0.001	0.76 (0.72–0.80); < 0.001	0.50 (0.46–0.55); < 0.001
60–69	*38,363*	0.88 (0.85–0.90); < 0.001	0.89 (0.85–0.93); < 0.001	0.68 (0.63–0.74); < 0.001
70–79	*40,865*	1.00 (reference)	1.00 (reference)	1.00 (reference)
80–99	*12,933*	0.96 (0.92–1.00); 0.06	1.09 (1.02–1.16); 0.01	1.47 (1.35–1.60); < 0.001
Patient sex				
Female	*87,219*	1.00 (reference)	1.00 (reference)	1.00 (reference)
Male	*81,628*	1.48 (1.45–1.51); < 0.001	1.34 (1.29–1.38); < 0.001	1.13 (1.07–1.20); < 0.001
Hierarchical symptom group				
1—Iron deficiency anaemia	22,738	0.87 (0.84–0.91); < 0.001	0.82 (0.77–0.87); < 0.001	2.14 (1.97–2.33); < 0.001
2—Anaemia	9715	0.92 (0.88–0.97); < 0.001	0.91 (0.84–0.98); 0.01	1.30 (1.16–1.47); < 0.001
3—Rectal bleeding	53,857	1.00 (reference)	1.00 (reference)	1.00 (reference)
4—Weight loss	10,135	1.04 (0.99–1.09); 0.10	0.97 (0.90–1.04); 0.35	1.08 (0.94–1.23); 0.28
5—Melaena	208	0.81 (0.60–1.10); 0.18	1.29 (0.85–1.96); 0.23	0.81 (0.35–1.89); 0.63
6—Change in bowel habit	32,846	1.02 (0.98–1.05); 0.32	0.94 (0.89–0.99); 0.02	1.03 (0.94–1.13); 0.51
7—Diarrhoea	19,202	0.93 (0.90–0.97); < 0.001	0.81 (0.75–0.86); < 0.001	0.92 (0.81–1.03); 0.15
8—Constipation	6787	1.04 (0.98–1.10); 0.21	0.88 (0.80–0.96); 0.01	0.81 (0.68–0.97); 0.02
9—Abdominal pain	12,140	1.10 (1.05–1.15); < 0.001	1.05 (0.98–1.12); 0.17	1.01 (0.88–1.15); 0.91
10—Defecation disorder	518	1.11 (0.92–1.34); 0.30	1.15 (0.86–1.53); 0.34	1.39 (0.82–2.34); 0.22
11—Bloating	701	1.08 (0.91–1.27); 0.40	1.19 (0.93–1.52); 0.16	0.94 (0.53–1.66); 0.83
FIT group				
	33,796	1.00 (reference)	1.00 (reference)	1.00 (reference)
10–19.9	32,708	1.35 (1.30–1.40); < 0.001	2.19 (2.04–2.35); < 0.001	3.31 (2.62–4.19); < 0.001
20–29.9	15,755	1.41 (1.35–1.47); < 0.001	2.37 (2.18–2.57); < 0.001	4.44 (3.46–5.70); < 0.001
30–39.9	10,125	1.38 (1.31–1.45); < 0.001	2.68 (2.45–2.94); < 0.001	6.65 (5.17–8.57); < 0.001
40–49.9	7117	1.38 (1.31–1.46); < 0.001	3.00 (2.72–3.31); < 0.001	8.51 (6.58–11.01); < 0.001
50–99.9	17,493	1.27 (1.21–1.32); < 0.001	3.30 (3.06–3.56); < 0.001	10.20 (8.15–12.78); < 0.001
100–199.9	18,800	1.11 (1.07–1.16); < 0.001	3.83 (3.55–4.12); < 0.001	21.19 (17.07–26.30); < 0.001
200–299.9	15,709	1.11 (1.06–1.17); < 0.001	3.36 (3.10–3.65); < 0.001	36.49 (29.42–45.27); < 0.001
≥ 300	17,344	0.96 (0.91–1.00); 0.04	3.79 (3.51–4.09); < 0.001	46.13 (37.25–57.12); < 0.001
Endoscopist variation		0.23 (0.21–0.25)	0.41 (0.37–0.46)	0.25 (0.20–C0.30)

*Note:* Analysis restricted to those for whom a numerical FIT result was recorded. ORs mutually adjusted for age, sex, symptoms, FIT and endoscopist (as a random effect). Small number of ‘unknown’ patient sex combined with female for analysis.

Abbreviations: aOR, adjusted odds ratio; CI, confidence interval; FIT, faecal immunochemical test.

There were minimal associations between symptom groups and pathology. IDA remained independently predictive of CRC when compared to rectal bleeding (aOR 2.2 (95% CI, 2.0–2.3), *p* < 0.001), with a weaker effect for anaemia (aOR 1.3 (95% CI, 1.2–1.5), *p* < 0.001), but there were no significant associations with yield across other presenting symptoms.

After adjusting for FIT, age and symptoms, male sex was associated with increased detection of small polyps (aOR 1.5 (95% CI, 1.5–1.5)) and large polyps (aOR 1.3 (95% CI, 1.3–1.4)), with a more modest (albeit statistically significant) association with CRC (aOR 1.1 (95% CI, 1.1–1.2)).

**FIGURE 3:**
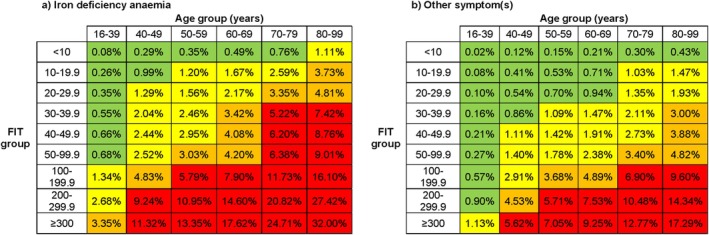
Modelled colorectal cancer yield (%) by age group and FIT concentration, stratified by (a) iron deficiency anaemia and (b) all other presenting symptoms. Colour‐coded thresholds: < 1% green, 1%–3% yellow, 3%–5% orange, > 5% red.

Modelled CRC yields were calculated separately for male and female patients, but as results were nearly identical (Table S2), sex was excluded from subsequent models. Although IDA was a stronger predictor of CRC than gastrointestinal symptoms, modelled outcomes demonstrated that in patients with FIT < 10 with IDA, the CRC yield from colonoscopy only exceeded 1% in those aged > 80 years. In contrast, IDA combined with FIT ≥ 10 was a strong predictor of CRC even in younger age groups, with risk increasing in parallel with FIT level (Figure 3a). For other presenting symptoms, the CRC yield remained below 0.5% across all age groups when FIT < 10 (Figure 3b). Yields with 95% confidence intervals are shown in Table S3.

Among 159,132 symptomatic colonoscopies with a recorded numeric FIT result for IDA or other symptoms, 4874 identified CRCs (3.1% (95% CI 3.0–3.1)), 9453 identified IBD (5.9% (95% CI, 5.8–6.1)), and 14,787 identified large polyps (9.3% (95% CI, 9.2–9.4)).

Had current guidance been followed, 25,743 (16.2%) colonoscopies would have been avoided, with 47 (1.0%) CRCs missed, alongside a low proportion missed of IBD and large polyps (Table 3).

**TABLE 3:** Comparison of current guidance and a risk‐stratified FIT model versus current practice for symptomatic colonoscopy.

		FIT threshold (μg Hb/g)		Impact versus current practice (*n*, % overall for model)			
Model	Age group (years)	IDA	Other symptom(s)	Capacity saved	Cancers missed	IBD missed	Large polyp(s) missed
Current Guidance	All ages	Investigate all	10	25,743 (16.2%)	47 (1.0%)	709 (7.5%)	988 (6.7%)
Risk‐stratified model	< 40	100	300	65,966 (41.5%)	263 (5.4%)	3397 (35.9%)	3505 (23.7%)
	40–69	10	30				
	≥ 70	Investigate all	10				

*Note:* ‘Missed’ pathology refers to cases not identified compared with current practice.

Abbreviations: IDA, iron‐deficiency anaemia; IBD, inflammatory bowel disease.

Applying a risk‐stratified model, in which colonoscopy was reserved for groups of patients with a CRC risk ≥ 1%, would have avoided 65,966 procedures (41.5%) while detecting 4611 CRCs (94.6%). However, this approach would have missed over a third of IBD cases (*n* = 3397; 35.9%) and nearly a quarter of large polyps (*n* = 3505; 23.7%).

## 4 Discussion

We show, for the first time at a national level, that FIT provides an effective means of triaging symptomatic colonoscopy referrals into those with high—and low—risk of significant pathology. Symptomatic colonoscopies undertaken following a negative FIT result had a very low likelihood of CRC detection and were three times less likely to identify IBD or large polyps, while the strong, graded association between FIT level and CRC demonstrates that FIT provides far greater predictive value than symptoms.

Previous NED analyses of symptomatic colonoscopy demonstrated variation in CRC yield by presenting symptom with a low overall yield of significant pathology [6]. Since the introduction of FIT triage, the diagnostic yield of CRC has increased by over 25% (1.9% vs. 1.5% in the earlier analysis), and the variations seen by sex and symptoms (other than IDA) with CRC yield have largely disappeared. However, only 34% of colonoscopies were performed in patients with FIT ≥ 10—where CRC yield was 3.8%—suggesting that further gains could be achieved through more widespread FIT utilisation and referral of fewer patients with FIT‐negative results, where CRC yield was just 0.3%.

The large dataset used in this analysis enabled more precise, stratified estimates of diagnostic yield by FIT level and age group than in previous regional studies [14, 15]. While CRC risk increased with age, a clear distinction emerged between patients aged 18–39, in whom risk remained very low, and those aged 40–49, whose risk was comparable to older cohorts; notably, the CRC yield for FIT ≥ 10 colonoscopies in the 40–49 age group was 2.5%. At a time when CRC incidence in those aged < 50 is rising [16], our findings highlight the potential value of low threshold FIT in identifying higher‐risk individuals in the 40–49 age cohort, but that below the age of 40 a high FIT threshold of at least 100 should be used.

Colonoscopy for IDA was associated with roughly double the CRC yield compared with procedures performed for other indications, consistent with previous studies [14, 15]. Building on this evidence, the COLOFIT project developed and validated a multivariable risk model incorporating FIT, age, sex, mean corpuscular volume (MCV) and platelet count, outperforming the current FIT ≥ 10 strategy for predicting colorectal CRC risk [17]. Our proposed pragmatic model—combining FIT, age and IDA status to guide symptomatic referral—would have detected over 94% of CRCs while reducing colonoscopy workload by more than 40%. However, the success of any such approach depends on universal FIT triage, which was recorded in only 45% of symptomatic colonoscopies in this national analysis.

Colonoscopies performed following a FIT ≥ 10 result were 2.5‐fold more likely to report IBD than those performed following FIT < 10, indicating that FIT has some discriminatory association with IBD detection. However, the yield of IBD among FIT < 10 colonoscopies remained substantial (2.6%), suggesting this discrimination is limited. This is reflected in the risk‐stratified model, in which strategies based on increasing FIT thresholds would be expected to miss over one‐third of IBD cases. Taken together, these findings indicate that while FIT may be associated with IBD detection, its utility for excluding IBD is limited and established biomarkers such as faecal calprotectin remain essential components of diagnostic pathways.

One of the concerns with FIT is its poor sensitivity for polyp detection. The current FIT ≥ 10 strategy misses over two thirds of polyps [18] and there is low sensitivity for advanced adenomas [19] and sessile serrated polyps [20]. We found FIT (both ≥ 10 and specific concentrations above this) had only a modest association with small polyps, but consistent with prior findings [20, 21], FIT did identify large polyps, with FIT ≥ 10 three times as likely to detect a large polyp as FIT < 10. These results indicate that FIT triage remains the most effective strategy available for identifying symptomatic patients with large polyps and that despite its limited sensitivity for polyp detection, its use should be encouraged to help reduce future CRC incidence, especially in a resource constrained system such as the NHS.

### 4.1 This Analysis Has Several Limitations

These mainly reflect the data source: although NED provides real‐world, national‐level data, it is currently anonymised, meaning it was not possible to link with other datasets such as pathology. This means our analysis could only be based on data from the endoscopy report (including the FIT result as entered by the endoscopist), meaning some indications and results may have been misclassified. This may result in misclassification of advanced neoplasia, particularly cancers arising within polyps that are not recognised as malignant at the time of endoscopy. However, misclassification would have had to be substantial, and differential, to account for the findings here and this seems unlikely. This limitation also prevented capture of diagnoses that rely mainly on histology, such as microscopic colitis. In addition, although more than 99% of UK sites contribute data to NED, some had not yet transitioned to NEDi2 during the study period so were not included in this analysis. However, there is no compelling reason to believe that findings would have been different in those sites which have not transitioned.

## 5 Conclusion

We have demonstrated, using real‐world national data relating to almost 450,000 procedures, the clear superiority of FIT‐based triage over symptom‐based referral for symptomatic colonoscopy. FIT distinguishes patients at highest risk of CRC and other significant pathology, while delineating a large group with minimal diagnostic yield. We demonstrate the value of a low threshold FIT (i.e., 10) down to the age of 40, but below this age, a high FIT threshold of at least 100 should be used.

To fully realise the benefits of this approach, integration of FIT into all symptomatic referrals should now be a national priority, alongside efforts to minimise colonoscopy in FIT‐negative patients. This approach would improve CRC detection, support equitable access to colonoscopy and ensure the greatest benefits for patients and the health service.

## Author Contributions

**David Beaton:** conceptualization, investigation, writing – original draft, writing – review and editing, methodology, formal analysis. **Mahmoud Mohamed:** conceptualization, writing – original draft, data curation.

## Funding

The authors have nothing to report.

## Conflicts of Interest

All authors have completed the ICMJE uniform disclosure form at http://www.icmje.org/disclosure‐of‐interest/. Linda Sharp has unrestricted project grants from 3D‐Matrix and Medtronic. All other authors declare: no support from any organisation for the submitted work; no financial relationships with any organisations that might have an interest in the submitted work in the previous 3 years; no other relationships or activities that could appear to have influenced the submitted work.

## Supporting information


**Table S1:** Supporting Information.

**Data S1:** Supporting Information.

**Data S2:** Classification of Indication and Diagnosis groups and free‐text matching process.

## Data Availability

The data that support the findings of this study are available from the corresponding author upon reasonable request.
