# Supplementary material for: Real‐World Performance of FIT Triage for Symptomatic Colonoscopy: Analysis of the UK National Endoscopy Database (NED)
Source: Aliment Pharmacol Ther. 2026 Jan 28;63(9):1297–305. doi: 10.1111/apt.70537 (PMC13089666; doi:10.1111/apt.70537)
Supplement: Supplementary file 1 — Table S1: Supporting Information. [file APT-63-1297-s002.docx]

*Supplementary Table 1. Classification of Indication and Diagnosis groups and free-text matching process*

1. Hierarchical indication groups with corresponding NED structured terms and free-text patterns used for classification.

| **Hierarchical Indication groups** | **NED Terms** | **Free-text Terms** |
| --- | --- | --- |
| 1 – Therapeutic | Stricture dilatation, planned polypectomy, tumour assessment, EMR. Also includes any procedures where therapy involved stent placement or removal, foreign body retrieval, balloon dilatation, argon plasma coagulation, or colonic decompression | None |
| 2 - Bowel Cancer Screening Programme (BCSP) | These cases were primarily identified using the structured fields: Referrer marked as “BCSP” and FIT value recorded as “Performed via BCSP” | Mentions of 'screen', 'bcs' |
| 3 - Abnormal prior investigation | Abnormality on Imaging modality , Abnormal sigmoidoscopy, bowelscope conversion, Abdominal mass, Rectal mass | Mentions of 'CTC', 'PET', 'scan', 'divertic', 'abnormal CT', 'bowelscope', 'conversion' |
| 4 - High risk cancer surveillance | High cancer risk surveillance, Post polypectomy surveillance, Polypectomy site check | Mentions of 'Lynch', 'post CRC', 'genetic', 'surveillance', 'polyp follow up', 'site ch' |
| 5 - Inflammatory bowel disease | IBD assessment, IBD surveillance, Elevated calprotectin | Mentions of 'colitis', 'Crohn', 'UC', 'proctitis', 'fcp', 'calpro', 'fc' |
| 6 – Symptomatic | Abdominal pain, Anaemia - other, Bloating, Change in bowel habit - fluctuating/variable, Harder or less frequent stool, PR mucus, Incontinence, Looser or more frequent stool, Iron deficiency anaemia, Melaena, PR Bleeding - altered blood, PR bleeding - anorectal, Weight loss | Mentions of 'pain', 'bloat', 'change', 'altered', 'cibh', 'consti', defecation', incontinence', 'mucus', 'loose', 'diarrhoea', 'ida', 'iron', 'bleed' |
| 7 – Only FIT recorded / No recorded indication | FIT positive, FOB positive, Blood-based multi-cancer screening | Mentions of 'fit+', 'positive', 'raised fit', Mentions of 'trial', 'research', 'fit<', 'fit neg', 'fit <' |

1. Diagnosis groups with corresponding NED structured terms and free-text patterns used for classification.

| **Diagnosis** | **NED Terms** | **Free-text Terms** |
| --- | --- | --- |
| Normal | Normal, Postoperative appearance, Melanosis | Mentions of 'postpolypectomy scar', 'surgical scar', 'emr scar', 'esd scar' |
| Diverticulosis | Diverticulosis | Mentions of 'divert' |
| Haemorrhoids | Haemorrhoids | Mentions of 'haemo', 'hemo' |
| Angioectasia | Angioectasia | Mentions of 'angio' |
| Rectal pathology | Anal fissure, Radiation proctopathy, Rectal ulcer, Varices | Mentions of 'fissure', 'radiation', 'varices' |
| Other diagnosis | Volvulus, Submucosal lesion, Stricture - postoperative, Stent occlusion, Stent in situ, Pneumatosis coli, Parasites, Lipoma, Foreign body, Fistula, Colitis - pseudomembranous, Colitis – ischemic | Mentions of 'luminal stricture', 'stricture - benign', 'lipoma', 'worm' |
| Polyp | Polyp, Polyposis syndrome | None |
| Inflammatory bowel disease (IBD) | Colitis - unspecified, Crohn's colitis, Crohn's - terminal ileum, Proctitis, Pouchitis, Stricture - inflammatory, Ulcerative colitis | Mentions of 'colitis', 'ulcer', 'erythema', 'ileitis', 'crohn', 'proctitis', 'inflammation' |
| Cancer | Colorectal cancer, Stricture – malignant | Mentions of 'malignant', 'cancer', 'crc' |
