# Supplementary material for: Real‐World Performance of FIT Triage for Symptomatic Colonoscopy: Analysis of the UK National Endoscopy Database (NED)
Source: Aliment Pharmacol Ther. 2026 Jan 28;63(9):1297–305. doi: 10.1111/apt.70537 (PMC13089666; doi:10.1111/apt.70537)
Supplement: Supplementary file 2 — Data S1: Supporting Information. [file APT-63-1297-s003.doc]

Supplemental Table 2. Modelled cancer yield (%) by age group, sex, and FIT group, stratified by (a) iron deficiency anaemia and (b) other presenting symptom(s). Colour-coded thresholds: <1% green, 1–3% yellow, 3–5% orange, >5% red.

|  | **Iron deficiency anaemia (female)** | | | | | | |  |  | **Iron deficiency anaemia (male)** | | | | | | |
| --- | --- | --- | --- | --- | --- | --- | --- | --- | --- | --- | --- | --- | --- | --- | --- | --- |
|  |  | **Age group (years)** | | | | | |  |  |  | **Age group (years)** | | | | | |
|  |  | 16-39 | 40-49 | 50-59 | 60-69 | 70-79 | 80-99 |  |  |  | 16-39 | 40-49 | 50-59 | 60-69 | 70-79 | 80-99 |
| **FIT group** | <10 | 0.08% | 0.28% | 0.34% | 0.48% | 0.75% | 1.10% |  | **FIT group** | <10 | 0.08% | 0.29% | 0.35% | 0.49% | 0.77% | 1.12% |
|  | 10-19.9 | 0.26% | 0.98% | 1.19% | 1.66% | 2.56% | 3.70% |  |  | 10-19.9 | 0.27% | 1.00% | 1.21% | 1.69% | 2.61% | 3.76% |
|  | 20-29.9 | 0.34% | 1.28% | 1.54% | 2.15% | 3.32% | 4.77% |  |  | 20-29.9 | 0.35% | 1.30% | 1.57% | 2.19% | 3.38% | 4.85% |
|  | 30-39.9 | 0.55% | 2.02% | 2.44% | 3.39% | 5.18% | 7.36% |  |  | 30-39.9 | 0.56% | 2.06% | 2.49% | 3.45% | 5.27% | 7.49% |
|  | 40-49.9 | 0.66% | 2.42% | 2.92% | 4.04% | 6.15% | 8.69% |  |  | 40-49.9 | 0.67% | 2.46% | 2.97% | 4.12% | 6.26% | 8.84% |
|  | 50-99.9 | 0.68% | 2.49% | 3.01% | 4.16% | 6.33% | 8.94% |  |  | 50-99.9 | 0.69% | 2.54% | 3.06% | 4.24% | 6.44% | 9.09% |
|  | 100-199.9 | 1.33% | 4.79% | 5.74% | 7.84% | 11.64% | 15.99% |  |  | 100-199.9 | 1.35% | 4.87% | 5.84% | 7.97% | 11.83% | 16.23% |
|  | 200-299.9 | 2.66% | 9.17% | 10.87% | 14.50% | 20.69% | 27.25% |  |  | 200-299.9 | 2.71% | 9.32% | 11.05% | 14.72% | 20.98% | 27.60% |
|  | ≥300 | 3.33% | 11.24% | 13.26% | 17.50% | 24.55% | 31.82% |  |  | ≥300 | 3.39% | 11.42% | 13.47% | 17.76% | 24.89% | 32.20% |
|  |  |  |  |  |  |  |  |  |  |  |  |  |  |  |  |  |
|  | **Other presenting symptom(s) (female)** | | | | | | |  |  | **Other presenting symptom(s) (male)** | | | | | | |
|  |  | **Age group (years)** | | | | | |  |  |  | **Age group (years)** | | | | | |
|  |  | 16-39 | 40-49 | 50-59 | 60-69 | 70-79 | 80-99 |  |  |  | 16-39 | 40-49 | 50-59 | 60-69 | 70-79 | 80-99 |
| **FIT group** | <10 | 0.02% | 0.11% | 0.14% | 0.19% | 0.27% | 0.39% |  | **FIT group** | <10 | 0.03% | 0.13% | 0.17% | 0.23% | 0.33% | 0.48% |
|  | 10-19.9 | 0.07% | 0.38% | 0.48% | 0.65% | 0.93% | 1.34% |  |  | 10-19.9 | 0.09% | 0.46% | 0.58% | 0.78% | 1.13% | 1.62% |
|  | 20-29.9 | 0.09% | 0.49% | 0.63% | 0.85% | 1.22% | 1.75% |  |  | 20-29.9 | 0.11% | 0.60% | 0.77% | 1.03% | 1.48% | 2.12% |
|  | 30-39.9 | 0.15% | 0.78% | 0.99% | 1.33% | 1.91% | 2.73% |  |  | 30-39.9 | 0.18% | 0.94% | 1.20% | 1.61% | 2.31% | 3.30% |
|  | 40-49.9 | 0.19% | 1.01% | 1.29% | 1.73% | 2.48% | 3.53% |  |  | 40-49.9 | 0.24% | 1.23% | 1.56% | 2.09% | 3.00% | 4.25% |
|  | 50-99.9 | 0.24% | 1.27% | 1.61% | 2.16% | 3.09% | 4.39% |  |  | 50-99.9 | 0.30% | 1.53% | 1.95% | 2.61% | 3.73% | 5.28% |
|  | 100-199.9 | 0.52% | 2.64% | 3.35% | 4.46% | 6.30% | 8.80% |  |  | 100-199.9 | 0.63% | 3.19% | 4.04% | 5.36% | 7.54% | 10.47% |
|  | 200-299.9 | 0.82% | 4.13% | 5.21% | 6.88% | 9.61% | 13.22% |  |  | 200-299.9 | 0.99% | 4.97% | 6.25% | 8.22% | 11.41% | 15.56% |
|  | ≥300 | 1.03% | 5.12% | 6.44% | 8.47% | 11.74% | 15.98% |  |  | ≥300 | 1.24% | 6.15% | 7.71% | 10.08% | 13.87% | 18.70% |
