# Supplementary material for: Real‐World Performance of FIT Triage for Symptomatic Colonoscopy: Analysis of the UK National Endoscopy Database (NED)
Source: Aliment Pharmacol Ther. 2026 Jan 28;63(9):1297–305. doi: 10.1111/apt.70537 (PMC13089666; doi:10.1111/apt.70537)
Supplement: Supplementary file 3 — Data S2: Classification of Indication and Diagnosis groups and free‐text matching process. [file APT-63-1297-s001.doc]

Supplemental Table 3. Modelled colorectal cancer yield (%) by age group and FIT concentration, stratified by (a) iron deficiency anaemia and (b) all other presenting symptoms. Yield (95% confidence interval)

|  | **a) Iron deficiency anaemia** | | | | | | |
| --- | --- | --- | --- | --- | --- | --- | --- |
|  |  | **Age group (years)** | | | | | |
|  |  | **16-39** | **40-49** | **50-59** | **60-69** | **70-79** | **80-99** |
| **FIT group** | **<10** | 0.08 (0.03 - 0.12) | 0.29 (0.17 - 0.40) | 0.35 (0.22 - 0.48) | 0.49 (0.32 - 0.65) | 0.76 (0.51 - 1.01) | 1.11 (0.72 - 1.49) |
|  | **10-19.9** | 0.26 (0.11 - 0.42) | 0.99 (0.67 - 1.31) | 1.20 (0.84 - 1.55) | 1.67 (1.25 - 2.09) | 2.59 (1.99 - 3.18) | 3.73 (2.82 - 4.63) |
|  | **20-29.9** | 0.35 (0.13 - 0.56) | 1.29 (0.82 - 1.75) | 1.56 (1.03 - 2.08) | 2.17 (1.52 - 2.82) | 3.35 (2.42 - 4.28) | 4.81 (3.44 - 6.18) |
|  | **30-39.9** | 0.55 (0.21 - 0.89) | 2.04 (1.30 - 2.78) | 2.46 (1.63 - 3.29) | 3.42 (2.41 - 4.43) | 5.22 (3.79 - 6.66) | 7.42 (5.35 - 9.49) |
|  | **40-49.9** | 0.66 (0.25 - 1.08) | 2.44 (1.52 - 3.36) | 2.95 (1.91 - 3.98) | 4.08 (2.79 - 5.36) | 6.20 (4.39 - 8.01) | 8.76 (6.21 - 11.32) |
|  | **50-99.9** | 0.68 (0.29 - 1.08) | 2.52 (1.76 - 3.27) | 3.03 (2.21 - 3.86) | 4.20 (3.27 - 5.13) | 6.38 (5.16 - 7.60) | 9.01 (7.20 - 10.82) |
|  | **100-199.9** | 1.34 (0.59 - 2.10) | 4.83 (3.55 - 6.11) | 5.79 (4.44 - 7.14) | 7.90 (6.48 - 9.33) | 11.73 (10.03 - 13.42) | 16.10 (13.64 - 18.56) |
|  | **200-299.9** | 2.68 (1.22 - 4.15) | 9.24 (7.02 - 11.46) | 10.95 (8.66 - 13.25) | 14.60 (12.35 - 16.86) | 20.82 (18.33 - 23.32) | 27.42 (24.10 - 30.73) |
|  | **≥300** | 3.35 (1.55 - 5.16) | 11.32 (8.70 - 13.94) | 13.35 (10.69 - 16.02) | 17.62 (15.06 - 20.18) | 24.71 (22.03 - 27.38) | 32.00 (28.42 - 35.57) |
|  |  |  |  |  |  |  |  |
|  | **b) Other presenting symptom(s)** | | | | | | |
|  |  | **Age group (years)** | | | | | |
|  |  | **16-39** | **40-49** | **50-59** | **60-69** | **70-79** | **80-99** |
| **FIT group** | **<10** | 0.02 (0.01 - 0.03) | 0.12 (0.08 - 0.16) | 0.15 (0.11 - 0.20) | 0.21 (0.15 - 0.27) | 0.30 (0.21 - 0.39) | 0.43 (0.30 - 0.56) |
|  | **10-19.9** | 0.08 (0.06 - 0.10) | 0.41 (0.34 - 0.49) | 0.53 (0.44 - 0.62) | 0.71 (0.60 - 0.83) | 1.03 (0.87 - 1.19) | 1.47 (1.22 - 1.72) |
|  | **20-29.9** | 0.10 (0.07 - 0.13) | 0.54 (0.43 - 0.66) | 0.70 (0.56 - 0.83) | 0.94 (0.76 - 1.12) | 1.35 (1.09 - 1.60) | 1.93 (1.54 - 2.31) |
|  | **30-39.9** | 0.16 (0.12 - 0.21) | 0.86 (0.68 - 1.03) | 1.09 (0.88 - 1.31) | 1.47 (1.19 - 1.75) | 2.11 (1.71 - 2.50) | 3.00 (2.41 - 3.59) |
|  | **40-49.9** | 0.21 (0.15 - 0.28) | 1.11 (0.88 - 1.35) | 1.42 (1.13 - 1.71) | 1.91 (1.53 - 2.28) | 2.73 (2.20 - 3.26) | 3.88 (3.10 - 4.66) |
|  | **50-99.9** | 0.27 (0.20 - 0.34) | 1.40 (1.19 - 1.60) | 1.78 (1.54 - 2.01) | 2.38 (2.08 - 2.68) | 3.40 (3.00 - 3.80) | 4.82 (4.17 - 5.46) |
|  | **100-199.9** | 0.57 (0.44 - 0.70) | 2.91 (2.55 - 3.26) | 3.68 (3.29 - 4.07) | 4.89 (4.42 - 5.36) | 6.90 (6.30 - 7.50) | 9.60 (8.61 - 10.60) |
|  | **200-299.9** | 0.90 (0.70 - 1.11) | 4.53 (4.01 - 5.06) | 5.71 (5.14 - 6.28) | 7.53 (6.85 - 8.21) | 10.48 (9.63 - 11.32) | 14.34 (12.97 - 15.71) |
|  | **≥300** | 1.13 (0.88 - 1.39) | 5.62 (5.00 - 6.23) | 7.05 (6.40 - 7.71) | 9.25 (8.48 - 10.02) | 12.77 (11.83 - 13.70) | 17.29 (15.77 - 18.81) |
